# Biofilm Formation on Dental Implant Biomaterials by *Staphylococcus aureus* Strains Isolated from Patients with Cystic Fibrosis

**DOI:** 10.3390/ma14082030

**Published:** 2021-04-17

**Authors:** Anna Minkiewicz-Zochniak, Sylwia Jarzynka, Agnieszka Iwańska, Kamila Strom, Bartłomiej Iwańczyk, Marta Bartel, Maciej Mazur, Anna Pietruczuk-Padzik, Małgorzata Konieczna, Ewa Augustynowicz-Kopeć, Gabriela Olędzka

**Affiliations:** 1 Department of Medical Biology, Medical University of Warsaw, Litewska 14/16, 00-575 Warsaw, Poland; anna.minkiewicz@wum.edu.pl (A.M.-Z.); sylwia.jarzynka@wum.edu.pl (S.J.); kamila.strom@wum.edu.pl (K.S.); malgorzata.konieczna@wum.edu.pl (M.K.); 2 Department of Microbiology, National Tuberculosis and Lung Diseases Research Institute, Płocka 26, 01-138 Warsaw, Poland; a.iwanska@igichp.edu.pl (A.I.); e.kopec@igichp.edu.pl (E.A.-K.); 3 The Chair and Department of Oral Surgery, Medical University of Lublin, Karmelicka 7, 20-081 Lublin, Poland; dent.iwanczyk@gmail.com; 4 Department of Chemistry, University of Warsaw, Pasteura 1, 02-093 Warsaw, Poland; mbartel@chem.uw.edu.pl (M.B.); mmazur@chem.uw.edu.pl (M.M.); 5 Department of Pharmaceutical Microbiology, Centre for Preclinical Research and Technology, Faculty of Pharmacy, Medical University of Warsaw, Banacha 1B, 02-097 Warsaw, Poland; anna.pietruczuk-padzik@wum.edu.pl

**Keywords:** biomaterials, Ti-6Al-4V, CoCr alloy, zirconia, biofilm, *Staphylococcus aureus*, cystic fibrosis, SEM, AFM

## Abstract

Implants made of ceramic and metallic elements, which are used in dentistry, may either promote or hinder the colonization and adhesion of bacteria to the surface of the biomaterial to varying degrees. The increased interest in the use of dental implants, especially in patients with chronic systemic diseases such as cystic fibrosis (CF), is caused by an increase in disease complications. In this study, we evaluated the differences in the in vitro biofilm formation on the surface of biomaterials commonly used in dentistry (Ti-6Al-4V, cobalt-chromium alloy (CoCr), and zirconia) by *Staphylococcus aureus* isolated from patients with CF. We demonstrated that *S. aureus* adherence and growth depends on the type of material used and its surface topography. Weaker bacterial biofilm formation was observed on zirconia surfaces compared to titanium and cobalt-chromium alloy surfaces. Moreover, scanning electron microscopy showed clear differences in bacterial aggregation, depending on the type of biomaterial used. Over the past several decades, *S. aureus* strains have developed several mechanisms of resistance, especially in patients on chronic antibiotic treatment such as CF. Therefore, the selection of an appropriate implant biomaterial with limited microorganism adhesion characteristics can affect the occurrence and progression of oral cavity infections, particularly in patients with chronic systemic diseases.

## 1. Introduction

Dental implant treatment is important for the recovery process in patients with developmental and acquired dental defects. Dental implants directly improve the oral cavity function and esthetics as well as a patient’s speech and general well-being [1,2]. In recent times, medical professionals have increasingly used dental implant procedures for tooth replacement. Consequently, there has been an increase in the number of patients with systemic diseases requesting dental implants to replace their missing teeth [3,4]. However, the increase in demand for dental implants is also associated with an increase in complications associated with the procedure [5]. Therefore, the planning and execution of dental implant placement often requires an interdisciplinary collaboration with recognizing and determining the consequences of any ongoing systemic diseases, particularly acute and chronic infections caused by bacteria growing in structures referred to as biofilms [6]. Biofilm is defined as a complex interaction of unicellular organisms, free-floating bacterial aggregates or aggregates attached to a surface, embedded in a matrix of extracellular polymeric substances that they have produced [7].

Dental plaque is a perfect example of synergism between the bacteria and the host who provide a surface for bacterial adhesion. The structure of biofilms was also first described in dental plaques in the human body [6]. Based on recent reports, the biofilm that forms on dental biomaterials is the most important risk factor leading to the development of implant-related infections. A biofilm starts forming immediately upon the adherence of bacteria to an implant surface [8]. Chronic implant-related infections may be caused by the microenvironment created by the biofilm, wherein the bacteria tolerate antimicrobial drugs, over-the-counter antiseptic agents, and host immune response mechanisms [6,9,10,11]. Biomaterial-related infections, dental plaque formation, chronic wounds, periodontal disease, and pneumonia are particularly dangerous in patients with cystic fibrosis (CF) and dental implants [10,12,13,14].

To date, approximately 700 bacterial species that form biofilms in the oral cavity have been described, including over 400 species capable of adhering to teeth and dental implants, forming a biofilm on their surfaces, and growing in the gingival sulci and pockets [8]. Although a biofilm typically forms within 2 to 6 h, the conditions in the oral cavity and the presence of nutrients may decrease the time needed for biofilm formation (both on teeth and on dental implants) to 30 min [15]. Moreover, the biofilm composition in people with edentulous areas closely corresponds to the bacteria found in adjacent tissues [15,16].

*Staphylococcus aureus* has been reported to be a common cause of chronic and treatment-refractory infections in many patients with dental implants, particularly among those suffering from CF [17,18,19,20,21]. Additionally, infection-related implant failure is also commonly associated with. *S. aureus* colonization and biofilm formation [22], which involves multiple stages, including adherence, maturation, and dispersal of bacteria [23]. *S. aureus* biofilm growth is initially due to multiple interrelated physical, chemical, and biological processes. *S. aureus* adhesion to abiotic surfaces and eukaryotic cells occurs via cell wall proteins, which play a role in cell-to-cell cohesion, defined as inter-cell interactions, within the biofilm [20,24]. The cell wall proteins are also a major virulence factor in infections associated with implants made of various biomaterials [25,26].

Various ceramic and metal components of dental implants may either facilitate or hinder bacterial adhesion. Bacterial colonization and biofilm formation on biomaterials are directly associated with implant surface characteristics such as porosity, hydrophobicity, chemical composition, and shape [27]. Early stages of bacterial colonization involve the formation of a cell monolayer, followed by cell aggregation facilitated by the extracellular matrix produced, which contains various extracellular polymeric substances and extracellular DNA. The presence of extracellular DNA is a key factor for the adherence of cells during *S. aureus* and *S. epidermidis* biofilm formation [23,28,29,30]. In addition, lipopolysaccharides and exopolysaccharides also participate in bacterial cell–surface interactions [31]. All these factors lead to the formation of a three-dimensional biofilm whose structure is resistant to both environmental factors and to the neutrophils and macrophages from the host’s immune system. Compared to planktonic bacteria, the bacteria that are part of a biofilm are 100–1000 times more resistant to antibiotics and antimicrobial agents, which may be due to the genetic, metabolic, and physical properties of biofilms [17,18,32,33]. Planktonic cells can detach from the surface of mature biofilm and disperse in organism to continue the cycle of biofilm formation on other surfaces and niche [34,35].

Many biomaterials are suitable for use in the human body and include both synthetic and natural materials; for example, metals (stainless steel, cobalt alloys, titanium alloys), ceramics (aluminum oxide, zirconium dioxide or zirconia, calcium phosphate), and synthetic and natural polymers [36,37,38]. The main material used for dental implants is titanium (Ti), which is one of the key biomaterials due to its biocompatibility, bio-inertness, tensile strength, elasticity, and corrosion resistance [38,39,40], but the aesthetic problems can be found due to the metallic color of the titanium. Therefore, dental research has focused on discovering natural tooth-colored implant material that improves the aesthetic appearance of dental implants or implant-supported bridges [41,42].

*S. aureus* is an opportunistic pathogen associated with biofilms infections, which are a common cause of pulmonary infections, ventilator-associated pneumonia, mucosal surfaces, surgical site infections, and implanted medical devices [43]. Additionally, the absence of studies reporting on the efficacy of commercially used biomaterials for dental implants in patients with diseases that are chronic, systemic, or associated with changes in the microbiome composition made the purpose of this study. The goal was to determine biomaterials that could be recommended for use in patients with CF. Our study focused on *S. aureus*, since *S. aureus* infections are considered to be a burden for the healthcare system.

## 2. Materials and Methods

### 2.1. Reagents

All reagents were purchased from Sigma Merck KGaA (Darmstadt, Germany) unless stated otherwise. Dehydrated culture media were purchased from Merck (Merck Millipore, Darmstadt, Germany).

### 2.2. Bacterial Strain Conditions

This study investigated 33 clinical strains of *S. aureus* obtained from the sputum samples of patients with known CF treated at the Department of Microbiology, National Tuberculosis and Lung Diseases Research Institute (Warsaw, Poland). All study participants provided informed consent to collect samples. Cultures of *S. aureus* were grown in Columbia Blood Agar with 5% sheep blood (bioMérieux, Marcy-l’Etoile, France) and Chapman Agar (bioMérieux, Marcy-l’Etoile, France) at 37 °C for 24–48 h in aerobic atmosphere. Antimicrobial susceptibility testing was performed using the standardized disk diffusion method and gradient method with Mueller-Hinton Agar (Oxoid, France). The results were interpreted according to the European Committee of Antimicrobial Susceptibility Testing (EUCAST) [44] breakpoints against the following antibiotics: cefoxitin, gentamycin, tobramycin, ciprofloxacin, levofloxacin, erythromycin, clindamycin, tetracycline, trimethoprim/sulfamethoxazole, linezolid, teicoplanin, and vancomycin. All the strains were stored frozen at −70 °C in Lysogeny broth medium (LB) (Sigma-Aldrich, St Louis, MO, USA) with 2% glycerol prior to the study.

### 2.3. Biofilm Assay

The colorimetric assay of biofilms with crystal violet staining was performed as previously described [45]. Fresh bacterial suspensions were prepared in LB from overnight cultures. The wells of a sterile 96-well flat-bottomed polystyrene microplate (Kartell S.p.A., Milan, Italy) were filled with 230 µL of LB medium with 2% glucose. The negative control wells contained only the sterile broth. Twenty microliters of overnight grown bacterial culture was adjusted to an optical density (OD) 600 of 0.1 (~10^7^ colony-forming units (CFU)/mL^−1^) and was added into each well. After 48 h, media was aspirated and the wells were washed with sterile water. The wells were then stained with 1% crystal violet for 15 min. The unbound crystal violet stain was then removed, and the wells were washed with sterile water, air-dried, and crystal violet was dissolved by addition 200 µL of 96% ethyl alcohol. The OD of each well was measured at 584 nm wavelength using an automated Synergy HTX multi-mode reader (BioTek Instruments Inc., Winooski, VT, USA). Based on the OD, the strains were categorized as: non-biofilm producers, weak, moderate, or strong biofilm producers, as previously described and standardized [46,47]. All tests were performed three times in five replicates, the results are presented as the mean and standard deviations; statistical analyses were performed using StatSoft Statistica 10.0 (licensed to the Medical University of Warsaw).

### 2.4. Preparation of Biomaterial Discs

Following standard dental laboratory procedures and the manufacturer’s recommendations, we manufactured polished, circular specimens 10 mm in diameter and 2 mm in thickness from titanium alloy Ti-6Al-4V (grade 5 titanium), zirconium dioxide (yttria-stabilized tetragonal zirconia polycrystals, 3Y-TZP), and cobalt-chromium (CoCr) alloy (Duceralloy C, DeguDent GmbH, Hanau, Germany), which are commonly used in clinical practice. Original materials were obtained from Silesia Dental (Środa Śląska, Poland).

#### 2.4.1. Biomaterial Discs Preparation

The discs were cleaned in an ultrasonic bath (POLSONIC, Warsaw, Poland) in the solution of 1% Extran^®^AP 15 (Merck KGaA, Darmstadt, Germany) and 99% deionized ultra-pure water (HYDROLAB, Straszyn, Poland) for 15 min, then rinsed three times in ultrapure water and ultrasonic bath for 15 min and next rinsed in ethanol 99.8% (Avantor, Gliwice, Poland) for 15 min. The discs before biofilm formation were treated by dry-heat sterilization.

#### 2.4.2. Surface Analysis

The micro-level surface topography of the biomaterials Ti-6Al-4V, cobalt-chromium alloy (CoCr), and zirconia were characterized by using Multimode 5 Atomic Force Microscopy instrument (Veeco, New York, NY, USA) upgraded to Multimode 8 version (Bruker). The images have been acquired in ScanAsyst^®^ mode using dedicated silicone cantilevers. Three randomly selected regions/specimen were measured in total a mean number of 9 measurements per material were registered. AFM was used to analyze the surface roughness average of the specimens (Ra) at a micro-level (scan size: 15 × 15 µm^2^).

### 2.5. Bacterial Biofilm Formation on Biomaterial Discs

Overnight cultures of *S. aureus* were grown on Chapman agar plates for 24 h at 37 °C. A single colony of bacteria was inoculated into 10 mL of LB medium to grow statically overnight at 37 °C. The bacterial suspension (1 mL each) was then transferred to 500 mL of the appropriate medium LB and cultured in a shaker incubator at 37 °C until the bacteria grew up to the mid-exponential phase. At the next step, each inoculum was adjusted to the value 10^8^ CFU mL^−1^. The concentration of bacteria was determined using a spectrophotometer using an automated Synergy HTX multi-mode reader (BioTek Instruments Inc., Winooski, VT, USA). Sterile, dry-heat sterilized, titanium alloy, zirconia, and CoCr alloy disks were inserted separately into 500 mL of LB medium supplemented with 0.25% glucose in conical flasks. Then, the discs were inoculated with *S. aureus* to a final concentration of 10^5^ CFU/mL^−1^ and cultured for 48 h at 37 °C in a shaker incubator at 160 rpm. The disks were aseptically removed from the cultures, washed gently three times in 10 mL 1· phosphate-buffered saline buffer, 7.4 pH (PBS) (Gibco™, Life Technologies, Bleiswijk, The Netherlands) to remove non-adherent cells, and then transferred to 50 mL PBS buffer.

### 2.6. Biofilm Detachment from Surfaces and Assessment of Viability (CFU Counting)

To count bacterial cells attached to the surface of the biomaterial discs after biofilm formation, the discs were placed in 15 mL Falcon tubes (Falcon^®^ Conical Tubes, STEMCELL, Reynosa, Mexico) containing 10 mL saline buffer and vortexed twice at 30 g for 3 min to dislodge the adherent bacteria. Ten-fold serial dilutions until 10^−6^ were prepared in saline buffer. Spread-plating (double tests) was performed on Chapman agar plates using 100 µL of the undiluted and six dilution samples. The CFU were counted after incubation at 37 °C for 24 h.

### 2.7. Scanning Electron Microscopy (SEM)

The surfaces of the titanium alloy, zirconia, and CoCr alloy disks were examined for bacterial attachment using a scanning electron microscope. The disks were mounted on aluminum stubs and sputter-coated with gold-palladium. The samples were then examined using a Zeiss Merlin field-emission scanning electron microscope (Zeiss, Oberkochen, Germany).

### 2.8. Fluorescence Microscopy

The surfaces of the titanium alloy, zirconia, and CoCr alloy disks were examined for bacterial viability using fluorescence microscopy. The specimens were stained with the LIVE/DEAD kit according to the manufacturer’s instructions (LIVE/DEAD™ BacLight™ Bacterial Viability Kit, Molecular Probes, Art. No. L7012, Invitrogen, Molecular probes, Eugene, OR, USA) and examined using fluorescence microscopy (Nikon Eclipse LV 100).

### 2.9. Statistical Analyses

The results are presented as the mean with standard deviations. All data were statistically analyzed from three independent experiments using the Student’s *t*-test. Values of *p* < 0.05 were considered to indicate statistical significance. Statistical analyses were performed using StatSoft Statistica 10.0 (licensed to the Medical University of Warsaw).

## 3. Results

### 3.1. Drug Susceptibility Profiles

The strains were resistant to cefoxitin 3/33 (9%), gentamycin 5/33 (15.2%), tobramycin 6/33 (18.2%), ciprofloxacin 11/33 (33.3%), levofloxacin 5/33 (15.2%), erythromycin 22/33 (66.6%), clindamycin 18/33 (54.5%), tetracycline 4/33 (12.1%), and trimethoprim/sulfamethoxazole 1/33 (3%). However, all strains were sensitive to linezolid, teicoplanin, and vancomycin. Methicillin resistance was observed in 3/33 strains (9%). The antibiotic resistance phenotypes of each isolate determined using diffusion discs on agar are presented in Table 1.

### 3.2. Clinical Strains with Different Capacity for Biofilm Formation

**Figure 1:**
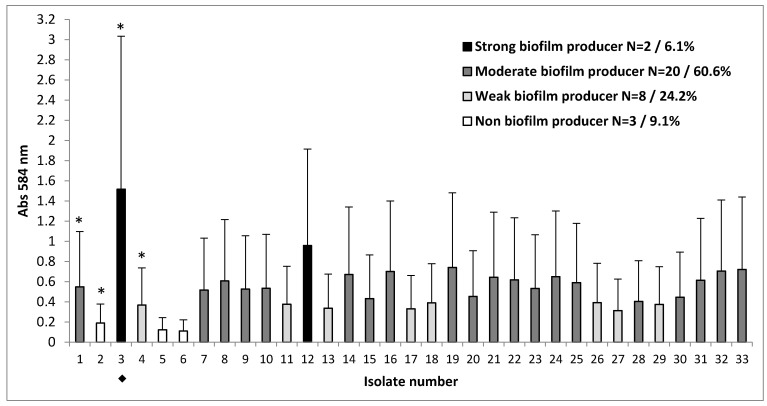
Biofilm-forming capacity of *S. aureus* clinical strains. The strains of *S. aureus* were assessed for their ability to form a biofilm and accordingly classified as strong, moderate, weak, and non-producers. Results are expressed as the mean value ± standard deviations of the mean of at least 5 independent experiments performed in triplicate. To classify the strains into groups that showed significant differences, statistical analysis was performed using Student’s *t*-test (* *p* < 0.05). The isolate number 3 that formed high levels of biofilm is indicated (◆).

We investigated biofilm formation of 33 clinical strains of *S. aureus* isolated from the sputum of patients with CF. To confirm the ability of the given *S. aureus* strains isolated from sputum to form biofilms, we used an in vitro test, as previously described. The capacity to form a biofilm after 48 h of incubation varied among the clinical strains of *S. aureus*. Based on their OD, the strains were categorized as strong, moderate, weak, or non-biofilm producers. Of the 33 clinical strains evaluated for ability of biofilm formation, nearly all strains (30/33; 90.9%) were capable of forming biofilms (Figure 1). As shown in Figure 1, 8/33 strains (24.2%) were categorized as weak, 20/33 (60.6%) as moderate, and 2/33 (6.1%) as strong biofilm producers. Student’s *t*-test showed significant differences in the amount of biofilm produced by these groups of strains (*p* < 0.05, *p* < 0.001).

Out of the 33 strains tested, one model strain of methicillin-resistant *S. aureus* (MRSA), *S. aureus* strain number 3 ◆ which was observed to be resistant to cefoxitin, tobramycin, ciprofloxacin, levofloxacin, erythromycin, and clindamycin and possessed strong biofilm-forming capacity, was chosen for further investigation.

### 3.3. Biofilm Formation in CFU

**Figure 2:**
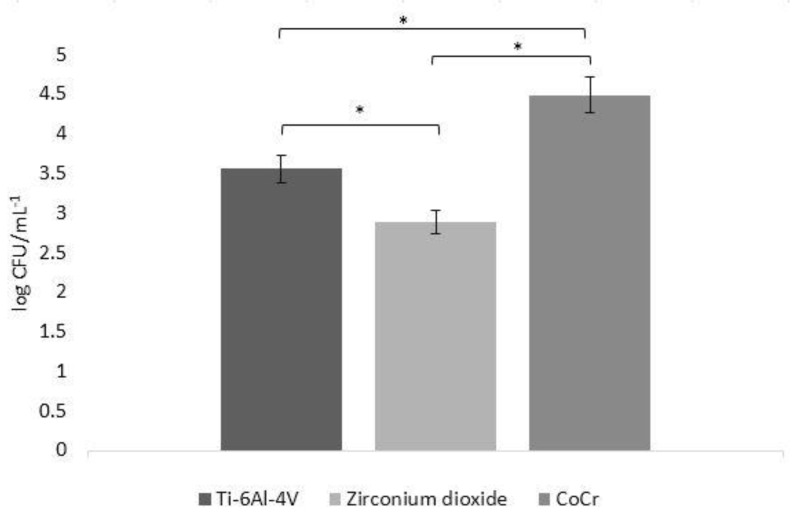
Biofilm formation (in colony-forming units (log CFU/mL-1)) on the surfaces of different biomaterials. Error bars represent the pooled standard deviations of the mean (*n* = 5). The level of significance was preset at * *p* = 0.05. The mean and standard deviation are shown.

The number of microbes adherent on biomaterial discs varied depending on the material used. *S. aureus* growth was significantly lower (*p* < 0.05) on the surfaces of zirconia compared to that on other materials. Biofilm density formed on the CoCr alloy surfaces was the highest, with significantly higher log CFU/mL^−1^ values, compared to those on the other evaluated materials (*p* < 0.05). Bacterial growth on Ti-6Al-4V surfaces was significantly lower (*p* < 0.05) than on CoCr alloy surfaces, but significantly higher (*p* < 0.05) than the growth on zirconia surfaces. Figure 2 shows the total biofilm formation (in CFU (log CFU/mL^−1^)) on the surfaces of different biomaterials.

### 3.4. Surface Topography Characteristics

**Figure 3:**
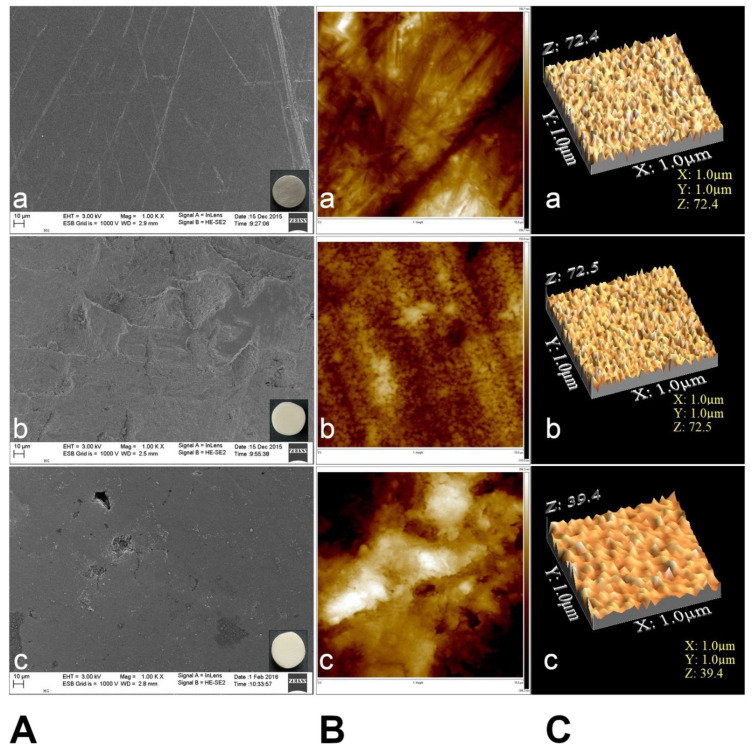
(**A**) Scanning electron microscopy (SEM) micrographs. Representative SEM images of the different biomaterial specimens. In all cases, the examined disks generally exhibited a smooth surface topography, with some fine polishing marks homogeneously distributed over the surface. (**a**), Ti-6Al-4V (grade 5 titanium); (**b**) zirconium dioxide (yttria-stabilized tetragonal zirconia polycrystals-3Y-TZP); (**c**) cobalt-chromium (CoCr) alloy (Duceralloy C). Original magnification ×1000 (Scale bar = 10 μm). (**B**) AFM micrographs show the surface topography of the tested biomaterials (**a**), Ti-6Al-4V; (**b**) zirconium dioxide; (**c**) cobalt-chromium (CoCr) alloy. Calibrated to 225 μm2 sample surface. (**C**) 3D atomic force microscopy (AFM) images for (**a**) Ti-6Al-4V; (**b**) zirconium dioxide; (**c**) cobalt-chromium (CoCr) alloy.

Representative baseline SEM images of the different biomaterial specimens are shown in Figure 3. In all cases, the examined discs exhibited a smooth surface topography, with a few fine polishing marks homogeneously distributed over the surface. Since all specimens were polished using a silicon carbide paper to achieve a highly glossy finish, the disks displayed similar surface topography upon examination.

AFM images exhibited differences in surface topography due to various biomaterials (Figure 3B,C). Polished zirconium surface (b) showed the lowest roughness values of Ra (23.8 ± 9.37 nm), while CoCr alloy showed the highest Ra (165.2 ± 79.80 nm) value. The titanium surface showed moderate values Ra (36.2 ± 15.25 nm). The Ti-6Al-4V and zirconium surfaces had parallel grooves along the polishing direction (Figure 3B(a,b)).

The mean values of surface roughness for the biomaterials are presented in Table 2.

### 3.5. Biofilm Formation by S. aureus on Three Different Biomaterials

**Figure 4:**
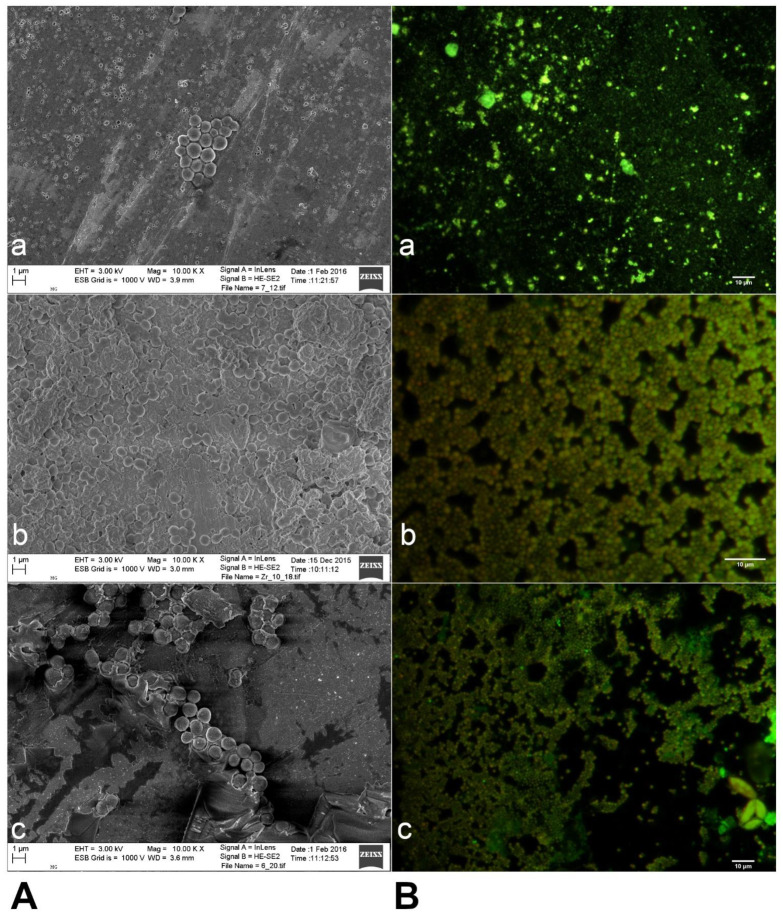
(**A**) Scanning electron microscopy images (**a**–**c**) of biofilms on different biomaterials. (**a**), Ti-6Al-4V (grade 5 titanium); (**b**) zirconium dioxide; (**c**) cobalt-chromium (CoCr) alloy (Duceralloy C). (**B**) Fluorescence microscopy images (**a**–**c**), after staining with a LIVE/DEAD BacLight kit on different biomaterials. (**a**) Ti-6Al-4V; (**b**) zirconium dioxide; (**c**) CoCr alloy (Duceralloy C). Original magnification ×10,000 (Scale bar = 1 μm).

One of the parameters that affect biofilm formation is the surface properties of the biomaterial. The biofilm production ability of one clinical *S. aureus* strain (strain number 3) was tested using three different biomaterials. Biofilms formed on all the tested biomaterial specimens. SEM images of the biofilm culture after incubation for 48 h are shown in Figure 4A. Figure 4B shows the fluorescence microscopy images obtained after staining the biofilms formed on Ti-6Al-4V, zirconia, and CoCr alloy surfaces with the LIVE/DEAD BacLight kit. Live bacteria cells in biofilm fluoresce bright green, whereas dead cells with compromised membranes fluoresce red-orange.

The microscopy images demonstrated that a greater number of bacteria aggregated on CoCr alloy compared to those on Ti-6Al-4V and zirconia surfaces (Figure 4A,B). After 48 h, the biofilm formed on the surface of zirconia was more densely colonized than that on Ti-6Al-4V and CoCr alloy surfaces. The biofilm colonies on the surface of Ti-6Al-4V were observed to be more scattered and horizontally spread than on the other examined biomaterials.

## 4. Discussion

Dental implant treatment with the use of selected biomaterials plays an important role in the convalescence of the oral cavity. Dental implants are regularly placed by dentists, and an increasing number of patients suffering from systemic diseases also opt for such implants to replace their missing teeth. However, it is important to understand and consider the consequences of pre-existing systemic diseases or diseases caused by chronic medications on the oral cavity. In patients with cystic fibrosis, observed impaired transport of ions and water transport in exocrine ducts of the salivary glands, modifying of the physical and chemical properties of saliva, and the qualitative and quantitative composition of microbiota inhabiting the oral cavity and affecting its health condition seems significant [48]. Such measures would help to prevent implant failures, especially in patients with CF who receive or plan to receive an implant treatment.

Due to the increasing prevalence of *S. aureus* in infections related to dental biomaterials and their occurrence in chronic and recalcitrant infections in patients with CF, this pathogen is considered an important healthcare problem [17]. In recent years, biofilms have increasingly been reported to play an important role in several human diseases and are responsible for numerous non-device-related chronic inflammatory conditions, including CF and biofilm-related inflammation [13]. The ability of clinical strains to form a biofilm is associated with the capacity of these organisms to survive in unfavorable conditions as well as on implanted biomaterials such as metals and ceramics [49]. Biofilm formation by *S. aureus* and its attachment to medical implants made of biomaterials and host tissue plays an important role in the persistence of chronic infections. Indeed, *S. aureus* has been reported to be the initial colonizer of dental implants [50].

The surface properties of biomaterials have determinant effects on biological behavior, and surface smoothness is of great importance in preventing the retention of microorganisms and biofilm formation. The results obtained in this study showed that the surface of zirconium was smoother compared to the CoCr alloy and Ti6Al4V (Ra), which correlated to the lower biofilm formation [51].

A prior study reported that bacteria in biofilms found in vivo engage in interspecies interactions, which may be important in the establishment of functional microbial communities in vitro [52]. Among the *S. aureus* strains isolated from sputum samples from patients diagnosed with CF, only 9% were unable to produce a biofilm in vitro, possibly because interspecies interactions might shape the development, structure, and functioning of these communities [53].

The ability of *S. aureus* to attach to dental biomaterial surfaces can result in oral diseases, periodontal disease, and dental caries caused by this microorganism. It may have a negative impact for health and therapy, especially in individuals with immunodeficiency, chronic systemic diseases, or chronic infection [54,55]. This study compared the susceptibility of three implant biomaterials towards *S. aureus* biofilm formation. *S. aureus* showed different potential for attachment to the various dental biomaterials assessed in this study. The bacterial attachment was dependent on factors such as surface roughness, electrostatic forces, and chemical composition of the biomaterials used. Therefore, investigation of biofilms grown on typical dental biomaterials plays an important role in achieving long-term success in combating implant-associated infections. An ideal implant material should be biocompatible, with adequate roughness, strength, and fracture resistance, as previously reported. [56]. Over the last 15 years, various forms of metal and ceramic coating have been used in clinical practice [57]. In this study, we investigated the most common dental implant materials made from metal (titanium and CoCr alloy) and ceramic (zirconium oxide) with similar surface roughness. However, the correlation between surface roughness and bacterial adhesion was not the subject of our research. Hence, the results of this study cannot be linked with specific surface morphologies.

The ability of *S. aureus* to form biofilms and their high in vitro affinity to titanium surfaces [16,58] and host tissues are important factors that promote chronic peri-implant infections, i.e., peri-implantitis. Moreover, *S. aureus* has been isolated from deep peri-implant pockets, where it presented with suppuration [40,58,59]. Indeed, the most persistent and recurrent infections are predominantly due to *Staphylococcus* spp., which have long been considered natural components of the oral microbiome. However, depending on the evaluated population and the associated comorbidities, the role of this bacteria in oral health is still a subject of debate [60]. *Staphylococcus* spp. are one of the key causes of peri-implant infections, and these ultimately lead to implant failure. However, no significant relationship between *Staphylococcus* infection and chronic periodontitis has been demonstrated [40,58,59].

Our in vitro study evaluated the differences in biofilm formation on the surfaces of three commercially available biomaterials commonly used in dentistry (Ti-6Al-4V, CoCr alloy, and zirconia), which is a limitation of the study. We report that *S. aureus* isolated from patients with CF can be reproducibly cultured on the surface of dental implant biomaterials. We also found that *S. aureus* adherence and growth depends on the type of material and its surface topography. To ensure reproducibility, all evaluated biomaterial samples were appropriately prepared, and their surfaces were identically polished to high gloss using silicon carbide paper. Baseline SEM scans showed smooth surfaces, with only a small number of shallow, homogeneous scratches that were a result of polishing. Prior studies have demonstrated that increased implant porosity and roughness may facilitate the adhesion of microorganisms to the implant surface [46,61,62]. Therefore, any part of the implant that is exposed to the oral cavity must be smooth to prevent plaque formation.

Bonsaglia et al. [63] have reported that enhanced bacterial adhesion and biofilm formation occurred on the surface of hydrophilic materials, including CoCr, when compared with hydrophobic materials, such as zirconia and titanium. Similarly, Jung-Su et al. [64] reported that *S. aureus* cells attached more easily to hydrophilic surfaces than to hydrophobic ones. The SEM images obtained in our study also showed clear differences in bacterial aggregation, with the CoCr alloy exhibiting increased bacterial aggregation and more uniform biofilm formation than Ti-6Al-4V and zirconia, which showed less cell-to-cell cohesion within the biofilm. The Ti-6Al-4V alloy surface showed more bacterial dispersal and a wider spread of bacterial growth than the surface of other biomaterials. A previous study had reported similar observations with fewer bacteria found attached to zirconia surfaces compared to titanium surfaces [65].

*S. aureus* biofilms formed on the CoCr alloy surface in our study were more aggregated than those formed on Ti-6Al-4V and zirconia (Figure 3A(a,b)). Similarly, Leonhardt et al. have reported lower bacterial adherence and density on titanium alloys [66]. We showed greater dispersion of the bacterial cells on titanium alloy than on zirconia and CoCr alloy. After culturing for 48 h, the biofilm on zirconia was more tightly colonized than those on Ti-6Al-4V and CoCr alloys. The uneven colonization of abiotic surfaces observed in this study is not uncommon for bacteria in biofilms.

Our findings suggest a more abundant biofilm formation on CoCr alloy than on either titanium or zirconia surfaces, which is consistent with the results of a prior study [67]. The in vitro studies on bacterial adhesion to various titanium and zirconium surfaces conducted by Rimondini et al. [65] and Grössner-Schreiber et al. [61] showed similar results, with considerably lower bacterial density found on zirconium than titanium surfaces. Scaraono et al. [68] also observed lower bacterial density fewer bacteria on zirconium oxide surfaces during the phase of adhesion. However, it has been suggested that bacterial density was due to the surface characteristics and electrical conductivity of zirconium oxide films. Moreover, several reports emphasized that due to their surface properties zirconia and zirconium nitrate can inhibit dental plaque formation on the implant and adjacent tissues, which may play an important role in soft-tissue healing and implant success [69]. More importantly, these biomaterials also help avoid peri-implant bone resorption [69]. On the other hand, Lima et al. [70] and Al-Ahmad et al. [66] reported that titanium and zirconia surfaces showed similar biological properties in terms of protein adsorption, biofilm composition, and bacterial adhesiveness.

In summary, our study indicated that zirconia is a better biomaterial for dental implants from a microbiological perspective, since less biofilm formation was observed on zirconia than on either CoCr alloy or titanium surfaces. A great number of reports on zirconia, demonstrating its lower potential for bacterial adhesion, has contributed to the increasing popularity of this biomaterial in orthopedics, in dentistry use for root canal posts, for prosthetic abutment, and in contemporary restorative dentistry [71]. Zirconia has been introduced in implant dentistry as an alternative to titanium, mainly due to its esthetic properties; the colour of natural teeth, absence of peri-implant tissue discoloration, and no visible metal abutments. Based on a three-year analysis, Balmer et al. [72] provided an additional reason for the preference for zirconia implants by patients. They argued that metals, such as titanium, undergo corrosion in the oral cavity due to electrochemical redox reactions, and this may cause hypersensitivity responses, as evidenced by the low, but significant, proportion of dental implant patients (0.6%) allergic to titanium.

In addition to the above factors, the potential for good osteointegration is of the utmost importance when selecting dental implants for patients in whom the risk of inflammation must be minimized due to their comorbidities. In patients with CF, the respiratory tract is profusely colonized by various bacteria, whose profile depends on both autogenic and allogenic factors. However, despite the scarcity of reports on patients with CF on the subject, *S. aureus* and *Pseudomonas aeruginosa* are found to be the predominant species, especially in adults [72]. These pathogens are also the most common causes of respiratory infections in patients with CF [73]. Over time, *S. aureus* strains have developed many mechanisms of resistance [74]. The intrinsic and mutation-mediated bacterial resistance to antimicrobial agents in conjunction with the lack of alternative effective treatment poses a challenge in treating infections. Studies on the oral cavity microbiome have demonstrated an increased resistance to both the immune mechanisms of the host and antimicrobial treatment, which may result in more severe and persistent infections in patients and contribute to the spread of resistant pathogens, thus increasing the risk on the patient’s health. Therefore, the development or selection of an appropriate implant biomaterial, with limited early microorganism adhesion, could limit the occurrence and progression of oral cavity infections, particularly in patients with CF.

In order to lower the risk of bacteria from developing resistance, it is also advisable to limit the use of antiseptic and antibiotic treatments in the initial stages of peri-implant infections in patients with CF. There is a need for further studies on non-invasive methods of cleaning dental implants and other elements used in tooth replacement reconstruction, with a particular focus on their impact on the surface and structure of biomaterials and their potential use in preventing biofilm formation.

## 5. Conclusions

Within the limitations of this study, the following conclusions were drawn: in the case of *S. aureus* biofilms, the number of bacterial cells and the density of the biofilm on zirconia was lower than that observed with Ti-6Al-4V and CoCr alloys. We further conclude that an important factor in the long-term success of implants is the selection of an appropriate biomaterial for the implant. In summary, through our in vitro studies, we have shown that there is a significant correlation between the dental biomaterials used and the amount of clinical *S. aureus* strains adhering to their surface and their ability to form a biofilm on their surface. Therefore, in clinical settings, implants with zirconia should be considered in patients with chronic infections, such as CF. Furthermore, long-term randomized clinical and microbiological studies should be conducted in the future to determine the in vivo benefits of zirconia in combating and preventing chronic biofilm infections.

## Figures and Tables

**Table 1:** Antibiotic resistance phenotype of the *Staphylococcus aureus* clinical strains.

Antibiotic/N of Strains *%*	Sensitive	Resistance
cefoxitin	30	3
	*91.0*	*9.0*
	Phenotype MSSA *	Phenotype MRSA **
gentamycin	28	5
	*84.8*	*15.2*
tobramycin	27	6
	*81.8*	*18.2*
ciprofloxacin	22	11
	*66.7*	33.3
levofloxacin	28	5
	*84.8*	*15.2*
erythromycin	11	22
	*33.3*	*66.7*
clindamycin	15	18
	*45.5*	*54.5*
linezolid	*33*	0
	*100*	
teikoplanin	33	0
	*100*	
vancomycin	33	0
	*100*	
tetracycline	29	4
	*87.9*	*12.1*
trimethoprim/sulfamethoxazole	32	1
	*97.0*	3.0

* MSSA—methicillin-sensitive Staphylococcus aureus. ** MRSA—methicillin-resistant Staphylococcus aureus.

**Table 2:** Surface roughness (Ra) of the tested biomaterials.

Casting Techniques and Alloys	Ra (nm) Mean ± SD *n* = 9
Ti-6Al-4V	36.2 ± 15.25
Zirconium dioxide	23.8 ± 9.37
CoCr alloy	165.2 ± 79.80

## Data Availability

Not applicable.
